# A Quantitative Deficiency in Peripheral Blood Vγ9Vδ2 Cells Is a Negative Prognostic Biomarker in Ovarian Cancer Patients

**DOI:** 10.1371/journal.pone.0063322

**Published:** 2013-05-23

**Authors:** Aurélie Thedrez, Vincent Lavoué, Benoit Dessarthe, Pascale Daniel, Sébastien Henno, Isabelle Jaffre, Jean Levêque, Véronique Catros, Florian Cabillic

**Affiliations:** 1 Unité Mixe de Recherche Institut National de la Santé Et de la Recherche Médicale 991, Université de Rennes 1, Rennes, France; 2 Faculté de médecine, Université de Rennes 1, Rennes, France; 3 Département de Gynécologie et d’Obstétrique, Centre Hospitalier Universitaire de Rennes, Rennes, France; 4 Service de Cytogénétique et Biologie Cellulaire, Centre Hospitalier Universitaire de Rennes, Rennes, France; 5 Département d'Anatomie et Cytologie Pathologiques, Centre Hospitalier Universitaire de Rennes, Rennes, France; 6 Département d’Oncologie Chirurgicale, Institut de Cancérologie de l’Ouest – René Gauducheau, Saint-Herblain, France; INSERM- CNRS- Univ. Méditerranée, France

## Abstract

Vγ9Vδ2 cells are cytotoxic T cells that are able to recognize epithelial ovarian carcinoma (EOC) cells. Therefore, Vγ9Vδ2 cell-based adoptive transfer is an attractive therapy for EOC. However, the inefficient *ex vivo* expansion after specific stimulation of Vγ9Vδ2 cells from some patients and the relationships between Vγ9Vδ2 cells and clinical course of EOC are issues that remain to be clarified. Herein, peripheral blood mononuclear cells (PBMCs) from 60 EOC patients were stimulated with bromohydrin pyrophosphate (BrHPP) or zoledronate, which are specific agonists of Vγ9Vδ2 cells. The compounds differed in their efficacies to induce *ex vivo* Vγ9Vδ2 PBMC expansion, but 16/60 samples remained inefficiently expanded with both stimuli. Interestingly, the Vγ9Vδ2 cells in these low-responding PBMCs displayed before expansion (*ex vivo* PBMCs) an altered production of the pro-inflammatory cytokines IFN-γ and TNF-α, a decreased naive fraction and a reduced frequency. No evidence of an involvement of CD4^+^CD25^+^Foxp3^+^ regulatory cells was observed. Importantly, our data also demonstrate that a Vγ9Vδ2 cell frequency of 0.35% or less in EOC PBMCs could be used to predict low responses to both BrHPP and zoledronate. Moreover, our data highlight that such a deficiency is not correlated with advanced EOC stages but is associated with more refractory states to platinum-based chemotherapy and is an independent predictor of shorter disease-free survival after treatment. These results are the first to suggest a potential contribution of Vγ9Vδ2 cells to the anti-tumor effects of chemotherapeutic agents and they strengthen interest in strategies that might increase Vγ9Vδ2 cells in cancer patients.

## Introduction

Human Vγ9Vδ2 cells are a predominant subset of peripheral blood γδ T cells that express a unique TCR with Vγ9-Vδ2 regions. These cells, which usually represent 0.5–10% of the peripheral lymphoid pool, react against various tumor cells through the recognition of phosphorylated isoprenoid derivatives defined as phosphoantigens [1], [2]. Vγ9Vδ2 cells can directly kill their targets and release pro-inflammatory cytokines that boost the anti-tumor effector cells of the adaptive immune system [3]. Due to these characteristics, the selective triggering of these cells could be of major interest in cancer immunotherapy [4]. Several currently available clinical-grade compounds are able to strongly activate Vγ9Vδ2 cells and, with IL-2, can induce the selective outgrowth of these cells *in vitro* and *in vivo*. These compounds are either synthetic phosphoantigens, such as bromohydrin pyrophosphate (BrHPP, Phosphostim™), or pharmacological inhibitors of the mevalonate pathway, such as the aminobisphosphonates (i.e., zoledronate, Zometa™). Such compounds have been recently assessed in passive or active immunotherapeutic trials in patients with hematological malignancies or solid tumors [5]–[14]. These treatments have been generally well tolerated and have induced encouraging objective responses in some patients [12], [15], [16].

Epithelial ovarian cancer (EOC) is the fifth most frequently occurring cancer in women and causes more deaths than any other gynecologic cancer. Most EOC patients are diagnosed at an advanced stage. Currently, all patients undergo surgical procedures, and 90% of patients also receive a platinum-based chemotherapy. However, the 5-year survival rate remains below 40%. Therefore, the increasing knowledge about the role of immunosurveillance in EOC has led to the exploration of innovative therapeutic strategies that target the immune system [17]. Recently, our group demonstrated that *in vitro* phosphoantigen-expanded Vγ9Vδ2 cells from EOC patients display high cytolytic activity against fresh ovarian autologous tumor cells, thus providing a rational for Vγ9Vδ2 cell-based adoptive transfer in EOC patients [18]. However, the relationships between Vγ9Vδ2 cells and progression or clinical outcomes of EOC remain unexplored. Additionally, some concerns exist about the efficacy of Vγ9Vδ2 cell expansions with conventional protocols that are based on the *ex vivo* stimulation of peripheral blood mononuclear cells (PBMCs) with a single dose of either BrHPP or zoledronate and culture conditions that require IL-2. These protocols are suitable for cells from healthy donors [19], [20]. However, they failed to efficiently expand the Vγ9Vδ2 cells from some EOC patients [18], similar to observations in other cancers [12], [14], [20]–[22]. It remains to be seen whether these failures in some EOC patients are related to intrinsic differences in the Vγ9Vδ2 cells or are due to differences in other environmental parameters. An understanding of such differences would help to optimize future clinical trials of Vγ9Vδ2 cell-based adoptive transfer therapies in EOC.

In this study, we investigated the following in a cohort of 60 EOC patients: the parameters associated with inefficient BrHPP- and zoledronate-induced Vγ9Vδ2 cell expansions and the possibility of an association between the presence of Vγ9Vδ2 cells and the clinical course of EOC. We report that PBMCs that were inefficiently expanded with BrHPP and with zoledronate have before expansion (*ex vivo* PBMCs) reduced frequencies of Vγ9Vδ2 cells and that these cells display alterations in their phenotype and functionality. In addition, we reveal that a Vγ9Vδ2 cell frequency of 0.35% or less in *ex vivo* EOC PBMCs predicts low responses to both BrHPP- and zoledronate-based stimulation protocols and that such a cellular deficiency is related to the clinical progression and recurrence of EOC after chemotherapy-based treatment.

## Results

### The Expansions of Vγ9Vδ2 PBMCs in Response to BrHPP and to Zoledronate are Lower in EOC Patients than in Healthy Donors

**Figure 1:**
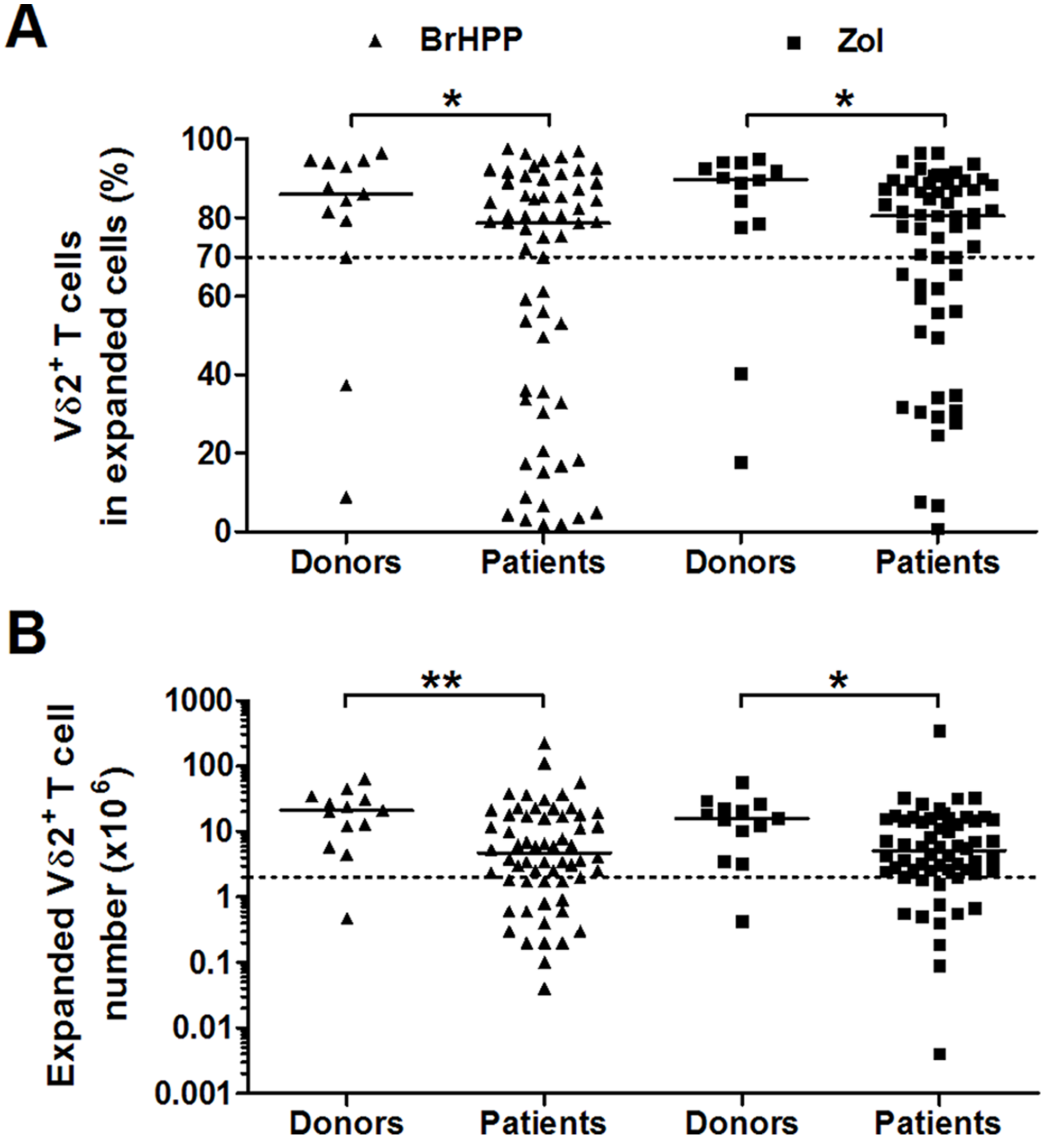
Proliferation of Vγ9Vδ2 PBMCs from EOC patients and from healthy female donors following BrHPP or zoledronate stimulation. Vδ2^+^CD3^+^ cell frequencies among expanded cells (**A**) and Vδ2^+^CD3^+^ cell numbers generated from 1×10^6^ PBMCs (**B**) were measured at 14 days after BrHPP (**▴**) or zoledronate (Zol) (**▪**) stimulation. The results from n = 60 patient PBMCs and n = 13 donor PBMCs are shown.

First, we compared the expansions of PBMCs from 60 EOC patients (EOC PBMCs) and from 13 healthy female donors after a specific Vγ9Vδ2 cell stimulation with a single dose of either BrHPP or zoledronate (Zol), which were relevant to clinical trial protocols, and a culture for two weeks in presence of IL-2 (Fig. 1). The median frequency of Vγ9Vδ2 cells in expanded PBMCs (Fig. 1A) and the median number of expanded Vγ9Vδ2 cells (Fig. 1B) were significantly lower in EOC patients than in donors at 14 days post-stimulation with either BrHPP or Zol. These data confirm that the responses of Vγ9Vδ2 PBMCs to both stimulation protocols are significantly reduced in EOC patients.

### BrHPP and Zoledronate Differ in their Capacities to Expand Vγ9Vδ2 PBMCs from EOC Patients

Taking into account our results from EOC patient PBMC expansions (Fig. 1) and the minimal rate of purity that was previously defined for a Vγ9Vδ2 cell-based therapy product [6], [23], we selected the expanded Vγ9Vδ2 cell number of 2×10^6^ cells (generated from 1×10^6^ PBMCs) (see Table 1 legend for details) and the Vγ9Vδ2 cell frequency of 70% among the expanded cells as cut-off values to distinguish efficient expansions (≥2×10^6^ cells and ≥70% ) from inefficient expansions (<2×10^6^ cells or <70%). Four statistically distinct groups of samples were thus identified in our experimental conditions (Table 1): the responding PBMCs (R) that were efficiently expanded with both stimuli (53%); the BrHPP-low-responding PBMCs (Br-LR) that were only efficiently expanded with Zol (13%); the Zol-low-responding PBMCs (Zol-LR) that were only efficiently expanded with BrHPP (7%); and the low-responding PBMCs (LR) that were inefficiently expanded regardless of the stimulus applied (27%). These data reveal the distinct abilities of conventional BrHPP- and zoledronate-based protocols to efficiently expand Vγ9Vδ2 PBMCs from EOC patients.

**Table 1:** Distinct expansions of Vγ9Vδ2 cells from 60 EOC patients in response to BrHPP and to Zol.

		Vδ2^+^ T cell frequency (%)			Vδ2^+^ T cell number (×10^6^)		
Groups	Nb. (%)	BrHPP	Zol	p value^$^	BrHPP	Zol	p value^#^
**All**	60 (100)	62+/−4	70+/−3	0.002	4.5 [1.5–17.5]	5 [2.5–15]	0.473
**R**	32 (53)	86+/−1	86+/−1	0.666	14 [6–22.5]	12 [4.5–17]	**0.026**
**Zol-LR**	4 (7)	77+/−2	56+/−4	<0.001	12.5 [2.5–22.5]	2 [0.5–13]	0.125
**Br-LR**	8 (13)	38+/−7	80+/−3	0.015	1.5 [1–3.5]	6 [3]–[12]	**0.015**
**LR**	16 (27)	20+/−5	35+/−5	<0.001	0.5 [0–2]	1.5 [0.5–3]	**0.022**

Vδ2^+^ T cell frequencies in expanded cells (Vδ2^+^CD3^+^ staining) and Vδ2^+^ T cell numbers generated from 1×10^6^ PBMCs were measured 14 days after BrHPP or zoledronate (Zol) stimulation; data are presented as means +/− SEM or medians with [first quartile - third quartile]; The Vδ2^+^ T cell frequency of 70% and the Vδ2^+^ T cell number of 2×10^6^ (corresponding to the mean of the first quartile values of expanded Vδ2^+^ T cell number with BrHPP and with Zol) were both used as cut-off values to distinguish efficient expansions (≥70% and ≥2×10^6^ cells) from inefficient expansions (<70% or <2×10^6^ cells); four PBMC groups were distinguished: R, Zol-LR, Br-LR and LR; Nb. (%) denotes the number of samples and the corresponding percentage; BrHPP and Zol data were compared using ^$^paired t-test or ^#^wilcoxon matched pairs test; p value <0.05 indicates a significant difference.

### The Capacities of Vγ9Vδ2 Cells to Proliferate and to Produce the Pro-inflammatory Cytokines IFN-γ and TNF-α are Reduced in LR EOC PBMCs

To investigate the parameters associated with the low-response status of Vγ9Vδ2 PBMCs to both BrHPP- and zoledronate-based stimulation protocols, comparative analyses were restricted to the LR and R groups.

**Figure 2:**
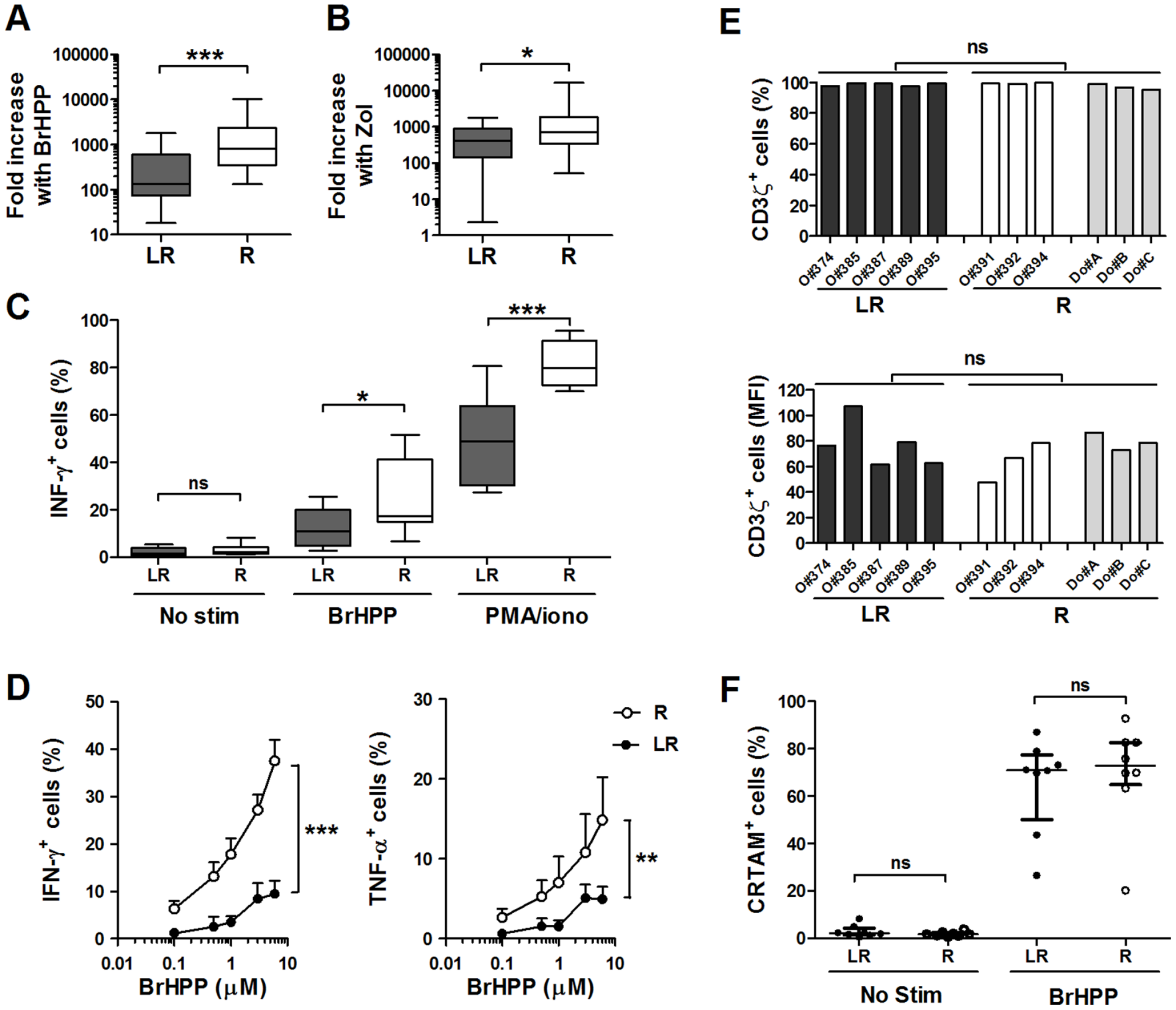
Reduced functionality of Vγ9Vδ2 cells in LR EOC PBMCs without defects in expression level of CD3ζ and CRTAM molecules. **A, B**) Vδ2^+^CD3^+^ cell fold increases in LR (n = 16) and R (n = 32) EOC PBMCs at 14 days after BrHPP (**A**) or Zol (**B**) stimulation. **C**) Percentages of IFN-γ^+^ cells in the Vδ2^+^CD3^+^ cells measured at 5 h after the stimulation of *ex vivo* LR (n = 10) and R (n = 10) PBMCs with BrHPP (3 µM) or PMA/ionomycin (PMA/iono). **D**) Percentages of IFN-γ^+^ cells (*left panel*) and TNF-α^+^ cells (*right panel*) among the Vδ2^+^CD3^+^ cells measured at 5 h after stimulations of *ex vivo* LR (n = 4) and R (n = 4) PBMCs with increasing doses of BrHPP (0.1 to 6 µM). **E**) Expression of the CD3ζ chain measured on Vγ9Vδ2 PBMCs from the LR and R groups. Percentage (*upper panel*) and MFI (*lower panel*) of CD3ζ staining in the Vδ2^+^CD3^+^ cells from LR EOC PBMCs (n = 5), R EOC PBMCs (n = 3) and R donor PBMCs (n = 3). **F**) CRTAM expression on Vγ9Vδ2 cells measured at 20 h after stimulation of LR (n = 8) and R (n = 8) EOC PBMCs with BrHPP. No stim: no stimulation.

We first compared the fold increases of Vγ9Vδ2 PBMCs 14 days after BrHPP or Zol stimulation (Fig. 2A and 2B). The fold increases were significantly lower in the LR PBMCs than in the R PBMCs after treatment with either agonist (Fig. 2A). The median BrHPP-induced Vγ9Vδ2 cell fold increase was 812 [352–2333] for the R group and only 132 [74–609] for the LR group (Fig. 2A). A similar trend was observed in Zol-treated cells, with median fold increases of 712 [339–1854] for the R group and 403 [140–872] for the LR group (Fig. 2B). The production of the pro-inflammatory cytokines IFN-γ and TNF-α byVγ9Vδ2 cells in LR and R EOC PBMCs before expansion (*ex vivo* PBMCs) was also addressed with intracellular staining. Experiments were performed by stimulating *ex vivo* PBMCs with BrHPP or PMA/Ionomycin. Due to few PBMC amounts in samples coming from patients, zoledronate was skipped from the following activation assays. The productions of TNF-α and IFN-γ were found to be significantly reduced in LR PBMCs as compared to R PBMCs (Fig. 2C and 2D). Importantly, doses of BrHPP that were saturating for R PBMCs failed to restore the IFN-γ and TNF-α responses in LR PBMCs (Fig. S1). All together, these results demonstrate an altered functional profile of Vγ9Vδ2 cells in LR EOC PBMCs.

The reduced proliferation and cytokine production of Vγ9Vδ2 cells could result from various factors including a reduced expression of T-cell receptor CD3ζ chains, altered proportions of naive and memory subsets or an unfavorable ratio of regulatory T cells to Vγ9Vδ2 cells. These possibilities were subsequently investigated.

### The Expression Level of CD3ζ Chains is Similar between the *ex vivo* LR and R EOC PBMCs

The activation signal transduction of T lymphocytes passes through the cytoplasmic tails of the T-cell receptor CD3ζ chains. A defect in the expression of these components on *ex viv*o Vγ9Vδ2 cells from LR PBMCs was explored. However, no significant differences were detected in the percentages and levels (MFI) of CD3ζ chain expression between *ex vivo* LR and R Vγ9Vδ2 PBMCs (Fig. 2E). We also compared the abilities of Vγ9Vδ2 cells in LR and R EOC PBMCS to express the class I-restricted T cell-associated molecule (CRTAM) [24], which was identified recently by our group as a phenotypic marker that is strongly associated with the activation of Vγ9Vδ2 PBMCs [25]. Interestingly, after BrHPP stimulation, CRTAM was found to be expressed similarly (percentage and MFI) on Vγ9Vδ2 cells from both LR and R PBMCs (Fig. 2F and data not shown).

### The Naive Vγ9Vδ2 Cell Fraction is Reduced in *ex vivo* LR EOC PBMCs

**Figure 3:**
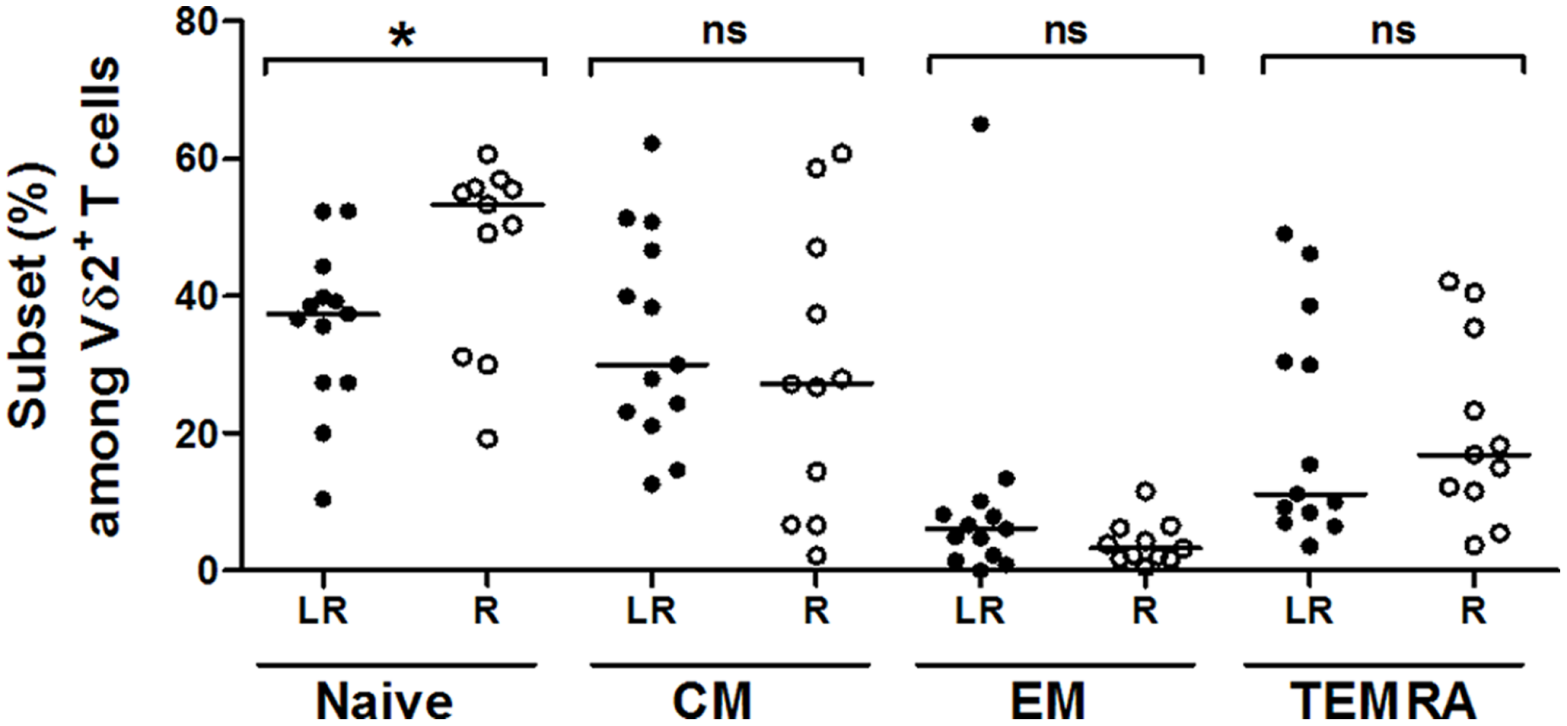
A reduced naive Vγ9Vδ2 subset in *ex vivo* LR EOC PBMCs. Percentages of naive (CD27^+^CD45RA^+^), central memory (CM) (CD27^+^CD45RA^−^), effector memory (EM) (CD27^−^CD45RA^−^) and terminally differentiated effector memory (TEMRA) (CD27^−^CD45RA^+^) cells among the Vδ2^+^CD3^+^ cells in *ex vivo* LR (n = 13) and R (n = 11) EOC PBMCs.

The naive and memory subsets of Vγ9Vδ2 cells were analyzed in *ex vivo* LR and R EOC PBMCs according to the expression of CD27 and CD45-RA markers (Fig. 3). The median naive cell frequency among Vγ9Vδ2 PBMCs was significantly lower in the LR group (37%) than in the R group (53%; Fig. 3). Accordingly, the median memory cell frequency was higher in the LR group. Significant positive correlation was found between the naive cell frequencies among Vγ9Vδ2 PBMCs and the fold increases of Vγ9Vδ2 cells after BrHPP stimulation (p = 0.041 according to the Spearman’s correlation test). Such correlation was not found in experiments performed with Zol. Within the memory compartment, no statistically significant differences were detected between the LR and R groups in the frequencies of central, effector or terminally differentiated effector subsets among Vγ9Vδ2 PBMCs (Fig. 3). Thus, no memory Vγ9Vδ2 subsets were enriched preferentially in the *ex vivo* LR EOC PBMCs.

### An Imbalance between CD4^+^CD25^high^FoxP3^high^ Regulatory T cells and Vγ9Vδ2 Cells Exists in *ex vivo* LR EOC PBMCs but is Not Involved in the Impaired Expansion of Vγ9Vδ2 Cells

**Figure 4:**
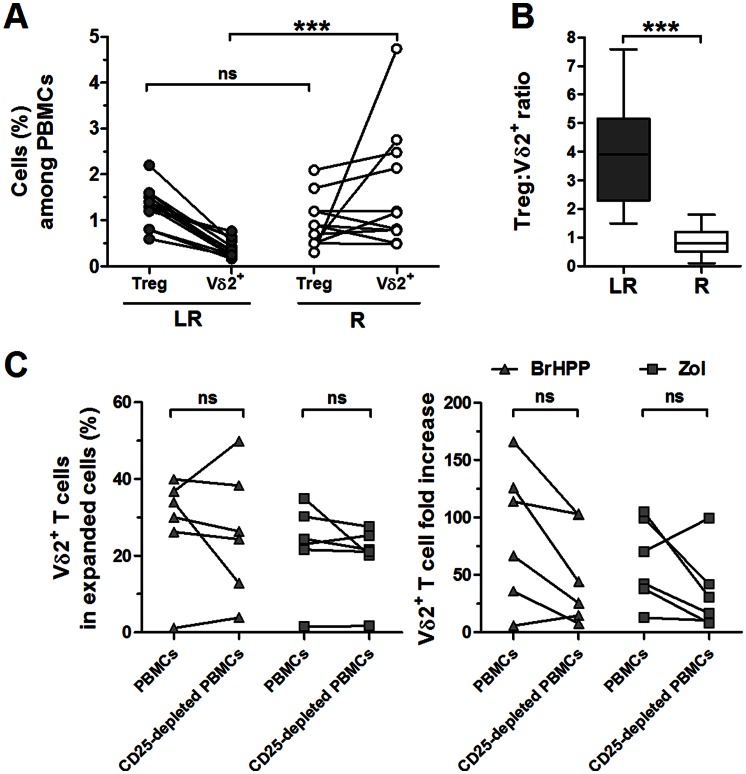
An imbalanced ratio of Tregs and Vγ9Vδ2 cells in *ex vivo* LR EOC PBMCs. **A**) CD4^+^CD25^high^FoxP3^high^ cell (Treg) frequencies and corresponding Vδ2^+^CD3^+^ cell frequencies in *ex vivo* LR (n = 13) and R (n = 11) PBMCs. **B**) The ratios of Tregs (%) to Vδ2^+^CD3^+^ cells (%) (Treg:Vδ2^+^ ratio) among PBMCs in the LR and R groups. **C**) LR PBMCs from EOC patients (n = 6) were stimulated with BrHPP (▴) or Zol (▪) and IL-2 before and after the depletion of CD25^+^ cells; the proliferation of Vδ2^+^CD3^+^ cells was analyzed on day 7. Vδ2^+^CD3^+^ cell frequencies among expanded cells (*left panel*) and Vδ2^+^CD3^+^ cell fold increases (*right panel*) are shown.

The frequencies of CD4^+^CD25^high^FoxP3^high^ regulatory T cells (Tregs) and the ratios of Tregs to Vγ9Vδ2 cells (Treg:Vγ9Vδ2 ratio) were explored in *ex vivo* LR and R EOC PBMCs. The Treg cell frequencies were not found to be significantly different between the LR and R EOC PBMCs (Fig. 4A). However, significant decreases in the Vγ9Vδ2 cell frequencies in LR EOC PBMCs led to Treg:Vγ9Vδ2 ratios that were significantly higher in the LR group than in the R group (Fig. 4B). Importantly, no inverse correlations were observed between these ratios and the fold increases of Vγ9Vδ2 cells that were measured after stimulation with either BrHPP or Zol (according to the Spearman’s correlation coefficient analyses, p = 0.07 and p = 0.67, respectively). Additionally, the depletion of Tregs in LR EOC PBMCs with anti-CD25 microbeads did not improve the expansion capacities of Vγ9Vδ2 cells after stimulation with BrHPP or Zol (Fig. 4C). Indeed, no significant differences were observed in the frequencies, numbers or fold increases of Vγ9Vδ2 cells from CD25-depleted LR PBMCs when compared to non-depleted LR PBMCs after stimulation with either BrHPP or Zol (Fig. 4C and data not shown). Of note, no depletions of Vγ9Vδ2 cells and altered cell viabilities were apparent after CD25-depletion (data not shown). Altogether, these observations suggest that the increased proportion of Tregs compared to Vγ9Vδ2 cells in LR EOC PBMCs is not involved in the impaired response of Vγ9Vδ2 cells.

### A Reduced Frequency of Vγ9Vδ2 Cells in *ex vivo* EOC PBMCs ≤0.35% Predicts an Inefficient Response to Both BrHPP and Zoledronate

**Figure 5:**
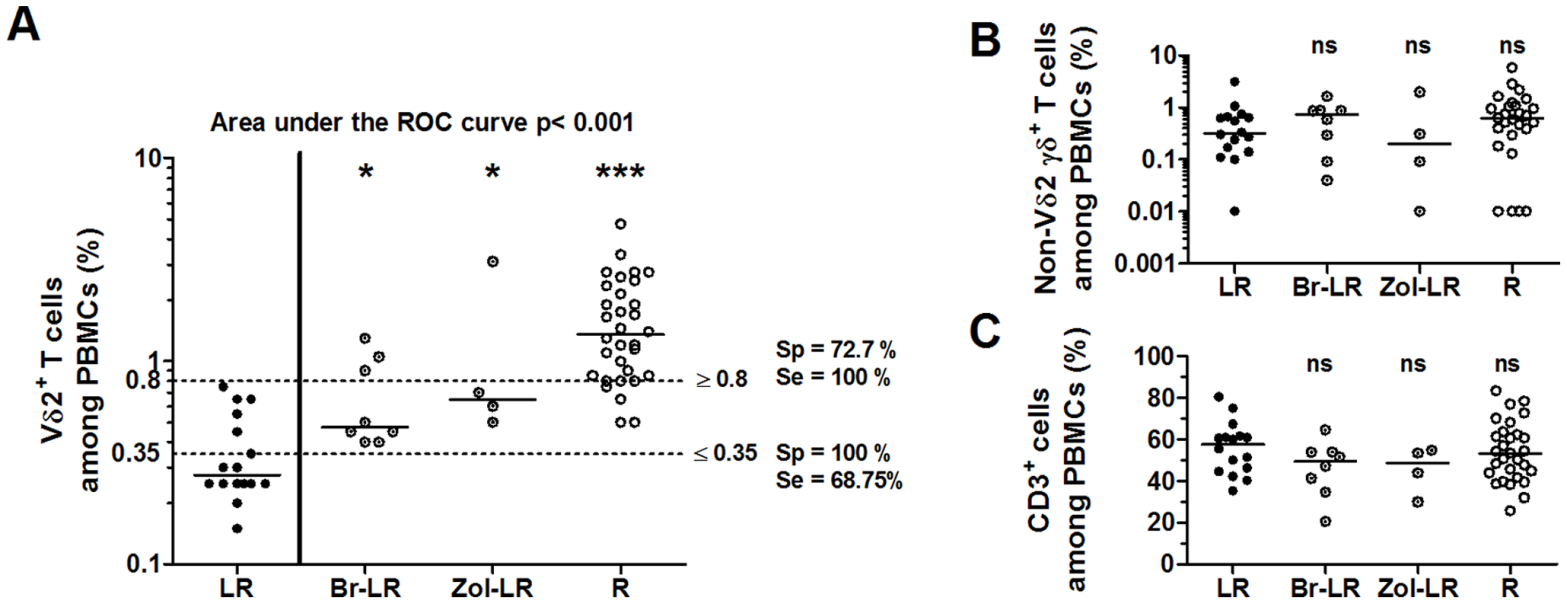
Specific quantitative deficiencies of Vγ9Vδ2 cells in *ex vivo* LR EOC PBMCs. Frequencies of Vδ2^+^CD3^+^ cells (**A**), Vδ2^−^γδ^+^CD3^+^ cells (**B**) and CD3^+^ cells (**C**) among LR (n = 16), Br-LR (n = 8), Zol-LR (n = 4) and R (n = 32) EOC PBMCs are shown. Differences between LR group and each of other groups are indicated. **A**) A receiver-operator characteristic (ROC) analysis was performed in which LR PBMC samples were compared to the other PBMC samples. Dashed lines indicate cut-offs at 0.35% and 0.8%. Sp: Specificity. Se: sensibility.

An analysis of the *ex vivo* Vγ9Vδ2 cell frequencies was then performed in the four groups previously described in Table 1. The frequency of Vγ9Vδ2 cells was significantly reduced in the LR group when compared to all other groups (Fig. 5A). The median values of Vγ9Vδ2 cell frequencies in PBMCs were 0.28% for the LR group and 1.38% for the R group (Fig. 5A). Intermediate values were observed for the Br-LR and Zol-LR groups, with median frequencies of 0.48% and 0.64%, respectively. Interestingly, no significant differences were observed between the LR, Br-LR, Zol-LR and R EOC groups in the frequencies of non-Vδ2^+^ γδ T cells (Fig. 5B) or of CD3^+^ cells in PBMCs (Fig. 5C). Besides, considering the whole cohort, a significant positive correlation was found between the *ex vivo* Vγ9Vδ2 cell frequencies and the fold increases after stimulation with BrHPP but not with Zol (p = 0.015 according to Spearman’s correlation tests). In addition, positive correlations between the *ex vivo* Vγ9Vδ2 cell frequencies and the IFN-γ-productions in response to either BrHPP or PMA/ionomycin (measured in Fig. 2C) were evidenced (p = 0.015 and p<0.001, respectively). These data reveal a specific reduction in the frequency of the Vγ9Vδ2 subset that is strongly associated with functional anomalies in LR EOC PBMCs.

To determine whether a particular Vγ9Vδ2 cell frequency value could be used to predict inefficient responses to both BrHPP and Zol, a receiver-operator characteristic (ROC) analysis was performed. A Vγ9Vδ2 cell frequency among PBMCs of 0.35% or less was always associated with low responses to both BrHPP and Zol (100% specificity). For frequencies of 0.8% or greater, all the expansions were efficient with at least one stimuli. For frequencies between 0.35% and 0.8%, inefficient responses to both stimuli and efficient responses were observed. These results demonstrate that a Vγ9Vδ2 cell frequency of 0.35% or less among *ex vivo* PBMCs is predictive of inefficient responses to both BrHPP and Zol.

### A Vγ9Vδ2 Cell Frequency of 0.35% or Less in *ex vivo* EOC PBMCs is a Negative Prognostic Marker in EOC Patients

To investigate the relationships between Vγ9Vδ2 cells and EOC disease, clinical data from patients were analyzed relative to the Vγ9Vδ2 cell frequencies in *ex vivo* PBMCs (Table 2 and Fig. 5). Among all the parameters that were recorded at the time of blood collection, an association was established only with patient age (Table 2). The median patient age was significantly higher in the group that had Vγ9Vδ2 cell frequencies in PBMCs of 0.35% or less (group≤0.35%) than in the group with frequencies greater than 0.35% (group>0.35%). Importantly, no decreases in the blood concentrations of leukocytes and PBMCs were observed in the group≤0.35%, which confirms the specific quantitative deficiency of Vγ9Vδ2 cells in the peripheral blood. No association was established between this deficiency and the administration of chemotherapy prior to blood collection. Moreover, no correlation was found between the deficiency in peripheral blood Vγ9Vδ2 cells and an advanced stage of disease or higher tumor grade. With regard to the treatment of EOC patients (Table 2), no significant differences were observed between the group≤0.35% and the group>0.35% in the efficacy of debulking surgery or in the type of received chemotherapy (Table 2). However, the clinical outcomes of chemotherapy-treated EOC patients were found to be distinct between the groups. Interestingly, the proportion of patients who were refractory to chemotherapy was significantly higher in the group≤0.35% (62.5%) than in the group>0.35% (17.5%) (Table 2). In addition, univariate analyses of disease-free survival with different relevant factors revealed that a Vγ9Vδ2 cell frequency of 0.35% or less in PBMCs and a non-optimal debulking are predictors of shorter disease-free survival duration (Table 3). With an average follow-up duration of 13 months for these patients, the median duration of disease-free survival was 1 month in the group≤0.35% versus 10 months in the group>0.35% (Fig. 5). Importantly, the negative prognostic value of the reduced Vγ9Vδ2 cell frequency was maintained after an adjustment in a multivariate analysis (Table 3).

**Table 2:** Clinical characteristics of EOC patients according to their Vδ2^+^ T cell frequency in PBMCs.

			≤0.35%	>0.35%	p value ^¥^
			n = 11	n = 49	
**At blood collection**	**Tumor histological type**	SP	7 (64)	36 (73.5)	0.712
		E	2 (18)	8 (16.5)	1.000
		M	2 (18)	1 (2)	0.084
		CC	0 (0)	4(8)	1.000
	**Tumor grade**	I	2 (20)	4 (9)	0.297
		II	5 (50)	12 (27)	0.254
		III	3 (30)	29 (64)	0.075
		nd	1	4	
	**FIGO stage**	I and II	2 (18)	9 (18.5)	1.000
		III	7 (64)	27 (55)	0.742
		IV	0 (0)	9 (18.5)	0.188
		recurrence	2 (18)	4 (8)	0.302
	**Ca125 (UI/ml)**	median [−]	203 [52–1042]	451 [51–1850]	0.607
	**Leucocytes (GIGA/L)**	median [−]	9.1 [5.6–10.4]	8.0 [6.3–10.3]	1.000
	**PBMCs (GIGA/L)**	median [−]	1.4 [0.9–2.4]	1.5 [0.9–1.9]	0.573
	**Blood punction after chemo**		4 (36)	9 (18)	0.231
	**Age (years)**	median [−]	**76 [70–78]**	**62 [54–71]**	**0.003**
**Treatment**	**Optimal debulking**	yes	6 (60)	25 (62.5)	1.000
		no	4 (40)	15 (37.5)	
		nd	1	9	
	**Platinum-based chemotherapy received**		9 (82)	43 (88)	0.630
	Carboplatin		2 (22)	5 (12)	0.590
	Carboplatin- taxol		6(67)	38 (88)	0.130
	Gemcitabine-oxaliplatin		1 (11)	0 (0)	0.173
	**Refractory to chemotherapy**	no	3 (37.5)	33 (82.5)	**0.016**
		yes	**5 (62.5)**	**7 (17.5)**	
		nd	1	3	

Patients with Vδ2^+^ T cell frequencies in PBMCs of 0.35% or less (≤0.35%) (n = 11) or greater than 0.35% (>0.35%) (n = 49) at the time of blood collection. Tumor histological type (serous-papillary (SP), endometrioid (E), mucinous (M), clear cell (CC)), tumor grade, FIGO stage, Ca125 concentration, leucocyte concentration, PBMC concentration and patient age at time of blood punction are indicated; PBMC samples collected after chemotherapy are noticed; the optimal debulking of tumor after surgery (no residual tumor nodules), the number of patients who received chemotherapy, the type of chemotherapy and the numbers of patients who were refractory to chemotherapy are indicated; data are presented as medians [first quartile – third quartile] for continuous variable and as numbers (with corresponding percentages) for qualitative variables; nd denotes case not determined;

¥ continuous variables were compared using the Mann-Whitney test and categorical variables using the Fisher’s exact test; p value <0.05 indicates a significant difference.

**Table 3:** Regression analyses of disease-free survival in surgery plus chemotherapy-treated patients.

	Univariate analysis				Mutivariate analysis Φ			
Variables	Nb	HR	95% CI	p value	Nb	HR	95% CI	p value
**Vδ2^+^ T cells in PBMC (%)**								
≤0.35	9	2.30	1.03 to 5.13	**0.042**	8	2.99	1.22 to 7.29	**0.016**
>0.35	43				43			
**Histologic type**					NI			
CC	4	1.71	0.52 to 5.59	0.378				
SP, E or M	48							
**Tumour grade** a					NI			
1	4	0.51	0.11 to 2.19	0.367				
2	15	1.28	0.62 to 2.64	0.496				
3	29							
**FIGO stage**					NI			
I or II	6	0.00	0 to 39.5E+168	0.947				
III	32	1.15	0.39 to 3.30	0.800				
IV	9	1.50	0.44 to 5.11	0.513				
Relapse	5							
**Optimal debulking** b								
no (>1 cm)	26	2.50	1.22 to 5.11	**0.012**	26	2.93	1.39 to 6.17	**0.005**
yes (≤1 cm)	25				25			
**Platinum-based chemotherapy**					NI			
without taxol	8	1.50	0.62 to 3.63	0.366				
with taxol	44							
**Age (years)**					NI			
≥70	21	1.20	0.61 to 2.35	0.596				
<70	31							

Results obtained using the Cox proportionnal hazard regression model; Nb denotes number of patients; HR denotes Hazard Ratio; CI denotes confidence interval; Tumor histological type (serous-papillary (SP), endometrioid (E), mucinous (M), clear cell (CC);

a data available in 48 patients;

b data available in 51 patients;

Φ multivariate analysis with data available in 51 patients : variables found to have a p value <0.1 in the univariate analysis were included in the model; NI denotes not included; p value <0.05 indicates a significant difference.

## Discussion

Previous studies have shown that conventional *ex vivo* Vγ9Vδ2 cell expansion protocols based on the stimulation of PBMCs with BrHPP or Zol failed in an average of 35% of cancer patients [12], [20]–[22], [26], [27]. Here, we confirmed these observations in EOC. From 60 EOC PBMC samples, inefficient expansions after stimulation with either BrHPP or Zol occurred in 40% and 33% of cases, respectively. Interestingly, our comparative analyses also revealed that BrHPP and Zol differed in their capacities to expand Vγ9Vδ2 PBMCs from patients. Samples that were not efficiently expanded with BrHPP could be efficiently expanded with Zol, and the reverse was also true. These results suggest that each compound might be tested in small-scale expansion assays to allow for the choice of the best compound for each patient. Nevertheless, 27% of PBMC samples from EOC patients remained inefficiently expanded with both compounds (LR PBMCs). We showed that the Vγ9Vδ2 cells in these *ex vivo* LR PBMCs not only had reduced proliferative capacities but also displayed an altered production of pro-inflammatory cytokines IFN-γ and TNF-α ?compared to the Vγ9Vδ2 cells from responding (R) PBMCs. Saturating doses of BrHPP failed to restore the response of Vγ9Vδ2 cells in LR PBMCs. These results support an intrinsic functional defect of LR Vγ9Vδ2 PBMCs and suggest that the use of higher doses of phosphoantigens to improve response of Vγ9Vδ2 cells in LR EOC PBMCs would be unsuccessful. To our knowledge, this is the first *ex vivo* demonstration of altered Vγ9Vδ2 cell effector functions in EOC patients.

Many parameters could be related to this reduced functionality. The loss or reduced expression of the CD3ζ chain on T cells from cancer patients has been implicated in impaired T cell activation [28]. However, no differences were observed in CD3ζ chain expression on *ex vivo* Vγ9Vδ2 cells between the LR and R PBMCs in our EOC cohort, which indicates that the expression of this chain is not causative. Defects in expression of activation markers could also be associated. Here, the molecule CRTAM, recently described by our group as a phenotypic marker that is strongly related with the activation of Vγ9Vδ2 PBMCs [25], was found to be expressed similarly on Vγ9Vδ2 cells from both LR and R EOC PBMCs after BrHPP stimulation. This observation suggests that signaling events leading to CRTAM expression are not defective in Vγ9Vδ2 from LR PBMCs. Besides, the presence of CD4^+^CD25^high^FoxP3^high^ T regulatory cells (Tregs) could be involved in the Vγ9Vδ2 cell deficiencies in cancer patients [29]. Recent data from Kunzmann *et al*. have documented that an increase in the Treg:Vγ9Vδ2 ratio suppressed phosphoantigen-induced γδ T cell proliferation and contributed to the state of apparent immunological unresponsiveness to phosphoantigens that was observed in some cancer patients [27]. Our results in EOC patients showed an increased Treg:Vγ9Vδ2 ratio in *ex vivo* LR PBMCs. However, we did not find an inverse correlation between this ratio and the fold increases of Vγ9Vδ2 cells in response to either BrHPP or Zol. Moreover, Kunzmann *et al*. have reported that the depletion of Tregs (using CD25-depletion) in low-responding PBMCs from patients with tumors others than EOC restored Vγ9Vδ2 cell expansion in response to phosphoantigens. Here, the same cell depletion in LR PBMCs from EOC patients did not improve the proliferation of Vγ9Vδ2 cells in response to phosphoantigens or aminobisphosphonates. In additional assays, we tested an oblique method of cell depletion to remove Tregs from LR EOC PBMCs while preserving CD25^+^ activated cells with the use of the CD4^+^CD25^+^CD127^dim/−^ Regulatory T Cell Isolation Kit II from Miltenyi Biotec (data not shown). Interestingly, effects of such depletion on the proliferation of Vγ9Vδ2 cells were similar to those observed with the CD25-depletion. All together, these results indicate that Tregs are not involved in the impaired proliferation of Vγ9Vδ2 cells in EOC.

Alternatively, differences in the *ex vivo* proportions of naive and memory Vγ9Vδ2 cell subsets could be associated with the reduced functionality of Vγ9Vδ2 EOC PBMCs, as it has been observed in other cancers [12], [21], [22]. Herein, we found that the naive subset was significantly reduced in *ex vivo* LR EOC PBMCs. This decrease might have been triggered by the repeated antigen priming of Vγ9Vδ2 cells *in vivo* during the development of the disease and with age, leading to a marked differentiation of naive cells to the memory cell compartment in some patients. The Vγ9Vδ2 cell frequencies in *ex vivo* PBMCs from patients could also be related to inefficient Vγ9Vδ2 cell expansions. Data from the literature on this issue are diverging. A correlation between the baseline percentages of Vγ9Vδ2 cells in PBMCs from different cancer patients and the *ex vivo* expansion capacities has been established recently [12], while another previous study reported no association between the same parameters in a large cohort of patients with different types of cancer [27]. Our study of EOC patients supports the most recent observation of specifically reduced Vγ9Vδ2 cell frequencies in *ex vivo* LR PBMCs. These deficiencies in Vγ9Vδ2 PBMCs, combined with decreases in proportion of the naive subset that is endowed with proliferative capacities [30], might be partly responsible for the reductions in Vγ9Vδ2 cell proliferation that were observed in some LR EOC PBMCs. Of note, the peripheral blood Vγ9Vδ2 cell frequencies in EOC patients were not affected by chemotherapy and we confirmed an age-dependent decrease of Vγ9Vδ2 cell frequencies in EOC patients, as was observed by other authors in healthy subjects [31], [32]. Importantly, we demonstrated that a Vγ9Vδ2 cell frequency among *ex vivo* PBMCs from EOC patients of 0.35% or less was always associated with inefficient expansions in response to BrHPP and to Zol. These data identify the Vγ9Vδ2 cell frequency in PBMCs of 0.35% or less as a unique biomarker that might be useful for the prediction of inefficient expansions of PBMCs from EOC patients in response to both conventional BrHPP- or Zol-based γδ expansion protocols.

Another important point addressed in this study was the relationships between Vγ9Vδ2 cells and the clinical outcomes of EOC patients who were treated with surgery plus platinum-based chemotherapy. Platinum derivatives have been described as immunomodulatory compounds, and candidate immune biomarkers such as αβ T cells have already been implicated in the efficacy of these anticancer agents [33], [34]. Two studies in human advanced ovarian carcinoma have reported that the presence of tumor-infiltrating CD3^+^ T cells is correlated with improved clinical outcomes of patients who were treated with surgery plus platinum-based chemotherapy [35], [36]. Of these two studies, one has also reported a correlation between the presence of infiltrating γδ T cells and a brief disease-free interval after treatment [36]. However, this study, which was based on a molecular assessment of TCRγ by PCR analysis, did not discriminate between Vγ9Vδ2 and other γδ cells. Here, we demonstrate that EOC patients with a decreased peripheral blood Vγ9Vδ2 cell frequency of 0.35% or less, which is correlated strongly with an impaired Vγ9Vδ2 PBMC functional profile, are more likely to be refractory to platinum-based chemotherapy and display a shorter disease-free survival time after treatment. A multivariate analysis confirms that this reduced frequency is an independent predictor for disease-free survival time. Therefore, the Vγ9Vδ2 cell frequency in PBMCs could be used as a prognostic biomarker in EOC patients. Taken together, our observations support the conclusion that the outcomes for chemotherapy-treated patients are more favorable when there are no deficiencies in the numbers and functionality of peripheral blood Vγ9Vδ2 cells. Interestingly, these data are the first to suggest that the Vγ9Vδ2 cell subset could contribute to the anti-tumor effects of conventional anticancer chemotherapeutics.

In conclusion, our results indicate that a specific Vγ9Vδ2 cell frequency value in *ex vivo* PBMCs could be used to select eligible EOC patients for conventional BrHPP- or Zol-based γδ expansion protocols and to predict the clinical outcomes for chemotherapy-treated EOC patients. The data suggest that the combination of current chemotherapeutic treatments in EOC with Vγ9Vδ2 cell-based immunotherapies could have a clinical interest for patients and should be explored. Our data also reveal that EOC patients with the worst prognostic outcomes are those with inefficient Vγ9Vδ2 PBMC expansions to both conventional BrHPP- or Zol-based γδ expansion protocols. Fortunately, other strategies that could increase Vγ9Vδ2 cells in these patients are conceivable. A powerful expansion protocol using aminobisphosphonates-pretreated dendritic cells or the transfer of allogenic Vγ9Vδ2 cells from healthy donors could represent alternative solutions [20].

## Materials and Methods

### Ethics Statement

This paper contains experiments using human cells. The paper satisfies PLOS ONE policies regarding human subject research. The Centre de Ressources Biologiques (CRB) from CHU of Rennes approved this study. The study was agreed by the french ministery of research and recorded under the n°"DC 2008-738". Written informed consent was obtained from all participating subjects.

### Cells from Donors and EOC Patients

PBMCs were isolated from peripheral blood samples from healthy female donors (Do#, n = 13 from the Etablissement Français du Sang, Villejean, Rennes, France) and patients with epithelial ovarian adenocarcinoma (EOC) (O#; n = 59 from the Gynecology Department, CHU of Rennes, France; n = 1 from Surgical Oncology, ICO-Cancer Center, Nantes; France) by the density separation method (Unisep®, Novamed, Jerusalem, Israel). Blood samples from EOC patients were collected before (n = 46) and after (n = 14) chemotherapeutic treatment. The mean age of the patients was 63.9 years (±SEM 1.5). Serous-papillary, endometrioid, mucinous and clear cell adenocarcinoma histological types were diagnosed anatomopathologically in 43, 10, 3 and 4 cases, respectively. Grades I, II, and III were noted in 11%, 31% and 58% of cases, respectively. The EOC staging was performed according to the International Federation of Gynecology and Obstetrics (FIGO) classification system [37]. The majority of the patients had advanced disease; 56.7% and 15% had FIGO stage disease III and IV, respectively, and 10% were in relapse. All patients underwent a surgical procedure. Platinum derivative-based chemotherapy was also performed for 52/60 patients. Carboplatin, carboplatin-taxol and gemcitabine-oxaliplatin agents were used in 7, 44 and 1 cases, respectively. Optimal debulking with surgery was defined by the absence of residual individual tumor nodules after surgery. A state refractory to chemotherapy was defined by increases in tumor size and/or CA-125 values at the end of the chemotherapy treatment. Refractory states were reported in 25% of the cases. The duration of disease-free survival was measured as the time between the end of treatment (surgery plus chemotherapy) and the first disease recurrence or progression. The duration of overall survival was calculated as the time between the end of treatment and death. Data were censored at the final follow-up for patients without disease recurrence, progression or death. The follow-up duration was defined as the time between the end of treatment and either death or the final follow-up.

### Antibodies and Reagents

Monoclonal antibodies (mAbs) against the following antigens were used for staining: Vδ2 (IMMU389), pan-γδ (IMMU510), CD45RA (ALB11), CD3 (CD3ε; UCHT1), CD3ζ (2H2D9), CD4 (13B8.2), CD14 (RMO52) and CD25 (B1.49.9) were obtained from Beckman Coulter (Villepinte, France); CD27 (o323) and Foxp3 (236A/E7) were obtained from eBioscience (Paris, France); CRTAM (210213) was obtained from R&D systems (Lille, France); and IFN-γ (B27) and TNF-α (MAb11) were obtained from BD Biosciences (Le Pont de Claix, France). Isotype-matched mAbs (Beckman Coulter, eBioscience, R&D systems or BD Biosciences) were used as staining controls. Synthetic BrHPP (Phosphostim™) was kindly provided by Innate Pharma (Marseille, France). Synthetic Zoledronate (Zometa®) was obtained from Novartis (Rueil-Malmaison, France). Recombinant human IL-2 (Proleukin®) was obtained from Chiron Therapeutics (Suresnes, France). Monensin, saponin, PMA and ionomycin were purchased from Sigma Aldrich (Saint-Quentin Fallavier, France).

### Vγ9Vδ2 Cell Expansion Assay

*Ex vivo* PBMCs were resuspended at 1.2×10^6^ cells/ml in RPMI 1640 medium (Eurobio, Les Ullis, France) that was supplemented with 10% fetal calf serum (FCS) (Gibco Invitrogen Life Technologies, Cergy Pontoise, France), 2 mM L-glutamine, 100 µg/mL streptomycin and 100 U/mL penicillin (hereafter referred to as RPMI-FCS) and were specifically activated with 3 µM BrHPP or 1 µM zoledronate plus 400 UI/ml recombinant IL-2. Cultures were maintained at 37°C for two weeks at 0.5×10^6^ cells/mL in RPMI-FCS plus 400 UI/ml IL-2. The concentrations of BrHPP, zoledronate and IL-2 that were used in this study were defined in accordance to clinical protocols [6], [12] and dose-scaling studies that ranged from 0.3 to 30 µM for BrHPP, from 1 to 10 µM for zoledronate and from 400 UI/ml to 3200 UI/ml for IL-2 (laboratory data). Vγ9Vδ2 cell specific expansion was measured by calculating the frequencies of Vδ2^+^CD3^+^ cells and the absolute Vδ2^+^CD3^+^ cell numbers at day 0 among PBMCs and at day 14 among expanded cells. The fold increases of Vγ9Vδ2 cells at day 14 were calculated according to the following formula: fold increase = (absolute Vδ2^+^CD3^+^ cell number at day 14)/(absolute Vδ2^+^CD3^+^ cell number at day 0).

### Depletion of CD25^+^ Cells and Vγ9Vδ2 Cell Proliferation Assay

*Ex vivo* PBMCs were depleted of CD25^+^ cells by a magnetic cell separation with CD25 microbeads (Miltenyi Biotec, Bergisch Gladbach, Germany) according to the manufacturer’s instructions. The efficacy of CD25 depletion in PBMCs and the frequency of Vδ2^+^CD3^+^ cells in depleted-PBMCs were evaluated by flow cytometric analysis. Next, CD25^+^-depleted PBMCs and non-depleted PBMCs from the same patients were resuspended at 1.2×10^6^ cells/ml in RPMI-FCS with 400 UI/ml IL-2 (0.6×10^6^ cells per well of a 48-well plate). The cells were stimulated by the addition of 3 µM BrHPP or 1 µM zoledronate and were incubated at 37°C for 7 days. At day 4, 0.25 ml of RPMI-FCS with IL-2 (400 UI/ml) was added to each well. The frequencies, numbers and fold increases of Vγ9Vδ2 cells at day 7 were measured as described previously.

### IFN-γ and TNF-α Responses and CRTAM Expression Assay

*Ex vivo* PBMCs were activated at 37°C in RPMI-FCS (1.2×10^6^ cells/ml) by the addition of BrHPP (0.1 to 30 µM) or PMA (20 ng/ml) plus ionomycin (0.5 µM). To measure IFN-γ and TNF-α responses, intracellular cytokine accumulation was induced by the addition of 3 µM monensin after 1 h of activation. The cells were collected 4 h later, stained for Vδ2 TCR chain and CD3 expression, and fixed with 1% formol. Fixation was followed by permeabilization with 0.5% saponin for 20 min and incubation with IFN-γ-specific or TNF-α specific mAbs for 30 min. To measure CRTAM expression, cells were collected 20 h after activation, stained for Vδ2 TCR chain, CD3 and CRTAM expression and fixed with 1% formol.

### Flow Cytometry and Statistical Analysis

**Figure 6:**
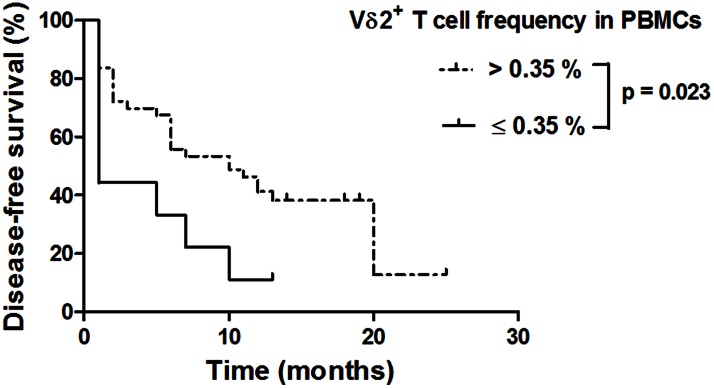
Disease-free survival of chemotherapy-treated EOC patients according to Vγ9Vδ2 cell frequencies among *ex vivo* PBMCs. Disease-free survival Kaplan-Meier curves of patients with Vγ9Vδ2 cell frequencies in PBMCs of 0.35% or less (≤0.35%) (n = 9) or greater than 0.35% (>0.35%) (n = 43) at the time of blood collection. p value <0.05 indicates a significant difference.

Stained cells were analyzed by flow cytometry with a FACSCalibur system (BD Biosciences) and FlowJo software (Tree Star, Ashland, OR, USA). Statistical analyses were performed with GraphPad Prism 5.0 (La Jolla, CA, USA) and Sigma plot 12.0 softwares (San Jose, CA, USA). The column scatter plots all show lines drawn at the medians. Comparison tests between groups were performed using Mann-Whitney U tests in Fig. 1, Fig. 2 (A, B, C, E and F), Fig. 3, Fig. 4 (A and B) and Fig. 5; a two-way ANOVA in Fig. 2D; a wilcoxon matched pairs test in Fig. 4C; and a log-rank (Mantel-Cox) test in Fig. 6. *, ** and *** indicate statistically significant differences for which p<0.05, p<0.01 and p<0.001, respectively. ns indicates that the difference was not significant.

## Supporting Information

Figure S1 **Reduced IFN-γ and TNF-α responses of Vγ9Vδ2** **cells in LR EOC PBMCs.** Percentages of IFN-γ^+^ cells and TNF-α^+^ cells among the Vδ2^+^CD3^+^ cells measured at 5 h after stimulation of *ex vivo* LR and R EOC PBMCs with increasing doses of BrHPP (n = 3). Comparison tests between groups were performed using a two-way ANOVA. *** indicates statistically significant differences for which p<0.001.
(TIF)
