# Supplementary figures and images for: A Quantitative Deficiency in Peripheral Blood Vγ9Vδ2 Cells Is a Negative Prognostic Biomarker in Ovarian Cancer Patients
Source: PLoS One. 2013 May 23;8(5):e63322. doi: 10.1371/journal.pone.0063322 (PMC3662688; doi:10.1371/journal.pone.0063322)

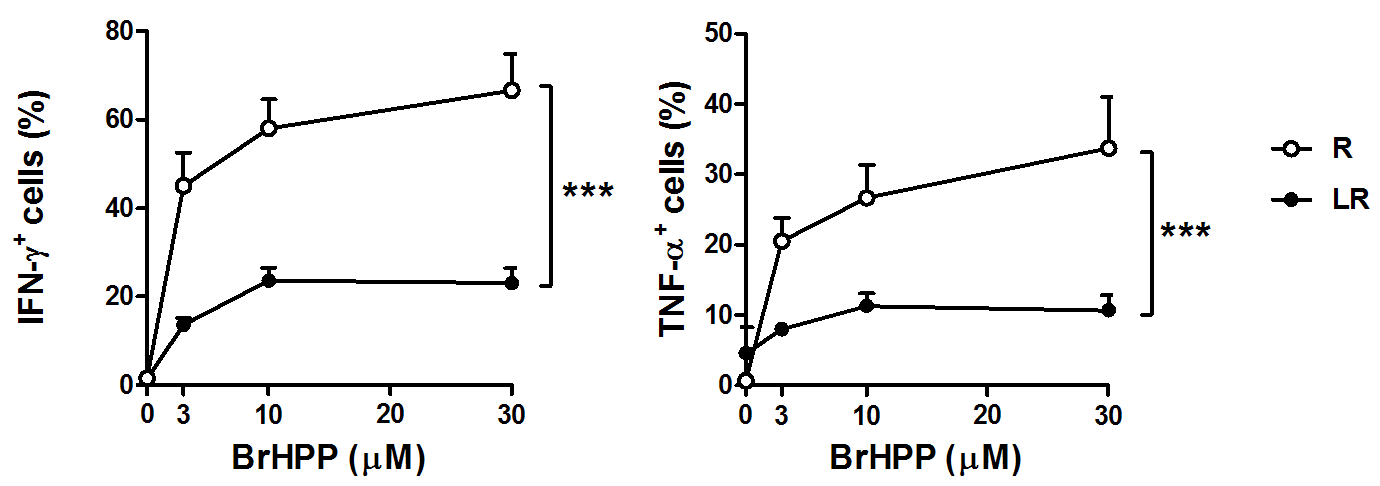

Supplement: Figure S1 — Reduced IFN-γ and TNF-α responses of Vγ9Vδ2 cells in LR EOC PBMCs. Percentages of IFN-γ+ cells and TNF-α+ cells among the Vδ2+CD3+ cells measured at 5 h after stimulation of ex vivo LR and R EOC PBMCs with increasing doses of BrHPP (n = 3). Comparison tests between groups were performed using a two-way ANOVA. *** indicates statistically significant differences for which p<0.001. (TIF) [file pone.0063322.s001.tif]
